# Cell cycle associated miRNAs as target and therapeutics in lung cancer treatment

**DOI:** 10.1016/j.heliyon.2022.e11081

**Published:** 2022-10-13

**Authors:** Atqiya Fariha, Ithmam Hami, Mahafujul Islam Quadery Tonmoy, Shahana Akter, Hasan Al Reza, Newaz Mohammed Bahadur, Md. Mizanur Rahaman, Md Shahadat Hossain

**Affiliations:** a Department of Biotechnology & Genetic Engineering, Noakhali Science and Technology University, Noakhali, Bangladesh; b Department of Genetic Engineering and Biotechnology, University of Dhaka, Dhaka, Bangladesh; c Department of Applied Chemistry and Chemical Engineering, Noakhali Science and Technology University, Noakhali, Bangladesh; d Department of Microbiology, University of Dhaka, Dhaka, Bangladesh

**Keywords:** miRNA, NSCLC, Cell cycle, Biomarker, Cancer therapy

## Abstract

Lung cancer is the primary cause of cancer related deaths worldwide. Limited therapeutic options and resistance to existing drugs are the major hindrances to the clinical success of this cancer. In the past decade, several studies showed the role of microRNA (miRNA) driven cell cycle regulation in lung cancer progression. Therefore, these small nucleotide molecules could be utilized as promising tools in lung cancer therapy. In this review, we highlighted the recent advancements in lung cancer therapy using cell cycle linked miRNAs. By highlighting the roles of the specific cell cycle core regulators affiliated miRNAs in lung cancer, we further outlined how these miRNAs can be explored in early diagnosis and treatment strategies to prevent lung cancer. With the provided information from our review, more medical efforts can ensure a potential breakthrough in miRNA-based lung cancer therapy.

CDK: A family of enzymes involved in regulation of the cell cycle along with Cyclins.

miRNA Mimic: Synthetic, double-stranded RNA molecules designed to mimic endogenous mature miRNAs.

AntagomiR: Synthetic single-stranded RNA molecules specially developed for inhibiting the expression of endogenous microRNA.

LNA:A class of high-affinity RNA analogs where the ribose ring is "locked" in the ideal conformation for Watson-Crick binding.

miRNA Sponges: Sponge RNAs offer complementary binding sites to a particular miRNA of interest, and are produced from transgenes within cells.

## 1 Introduction

According to GLOBOCAN (Global Cancer Incidence, Mortality and Prevalence), 2020, lung cancer is the second most frequently diagnosed malignancy and the foremost cause of cancer-related fatalities, representing approximately 2.2 million (11.4 %) new cases and 1.8 million (18 %) deaths worldwide [1]. Pathologically, lung cancer can be broadly subtyped into small cell lung cancer (SCLC) and non-small cell lung cancer (NSCLC). While SCLC may be more metastatic, 85% of the modern lung cancer cases are of NSCLC making it one of the most prevalent cancers at the time. This malignancy has become a major concern as the majority of the patients are diagnosed at advanced stages when the possibility of offering potentially curative surgical treatment is limited [2]. However, due to the molecular complexities of lung cancer and the development of drug resistance, the treatment failure rate is too high. Five-year survival rates are shown to be significantly improved when it is diagnosed at the early stage; unfortunately, the diagnosis rate at this phase is only 16% whereas the 5-year survival rate seems to be only 4% for the metastatic tumors in the case of advanced stages [3]. Although low-dose computed tomography (LDCT) screening is being used in high-risk individuals to detect cancer at an early stage, its application is restricted due to high false positive rates, exposure to potentially hazardous radiation, and the failure to distinguish indolent nodules from malignancies [4]. These challenges underline the crucial need for the validated biomarkers with high sensitivity and specificity for early lung cancer detection.

Abnormal cell proliferation is one of the major hallmarks of lung cancer. The precise regulation of the cell cycle is maintained by both cell-cycle progression and inhibition associated proteins. Furthermore, aberrant or dysfunction of miRNAs has been found to be involved in the cell cycle regulation of lung cancer [5, 6]. miRNAs and these cell cycle associated regulators interact in such a way that miRNAs control the expression of cell cycle regulators as either oncogenic or tumor suppressor miRNAs. Additionally, miRNAs can effectively suppress target genes while concurrently regulating a large number of genes of interest, which contributes to the treatment of cancer as a heterogeneous illness [7]. As a result, the identification and validation of effective role of miRNAs in cell cycle progression resulted in the emergence of novel treatment options for all forms of cancer progression.

Surgery, chemotherapy and radiotherapy are the most common traditional therapies used in case of lung cancer treatment [8]. However, these approaches fail to provide effective treatment for advanced cancer patients due to the involvement of various regulatory factors and genes in the development of resistance towards the processes. Hence, there is a great need for developing a more advanced and effective treatment approach that can eliminate tumor cells without any invasion or destruction of the normal cells. Furthermore, recent evidence suggests that aberrant function of miRNAs in lung cancers can also be involved in the resistance to both radiotherapy and chemotherapy [9, 10]. Therefore, we have tried to focus on the miRNAs involved in cell cycle regulation of lung cancer to highlight them as genomic medicine. This can be an emerging area as an individual therapy system or can be used in combination with radiation or chemical drugs based on their response towards these two traditional lung cancer therapies.

In this current review, we describe the role of miRNAs in the regulation of cell cycle as well as their association with lung cancer progression. In addition, we have tried to explore the most promising studies reported to investigate the clinical capability of miRNAs, either as biomarkers or therapeutics. We also highlighted the responsiveness of miRNAs towards chemotherapy and radiotherapy to be able to extend into suggesting a combinatorial therapeutic approach for lung cancer treatment.

## 2 Brief history

**Figure 1:**
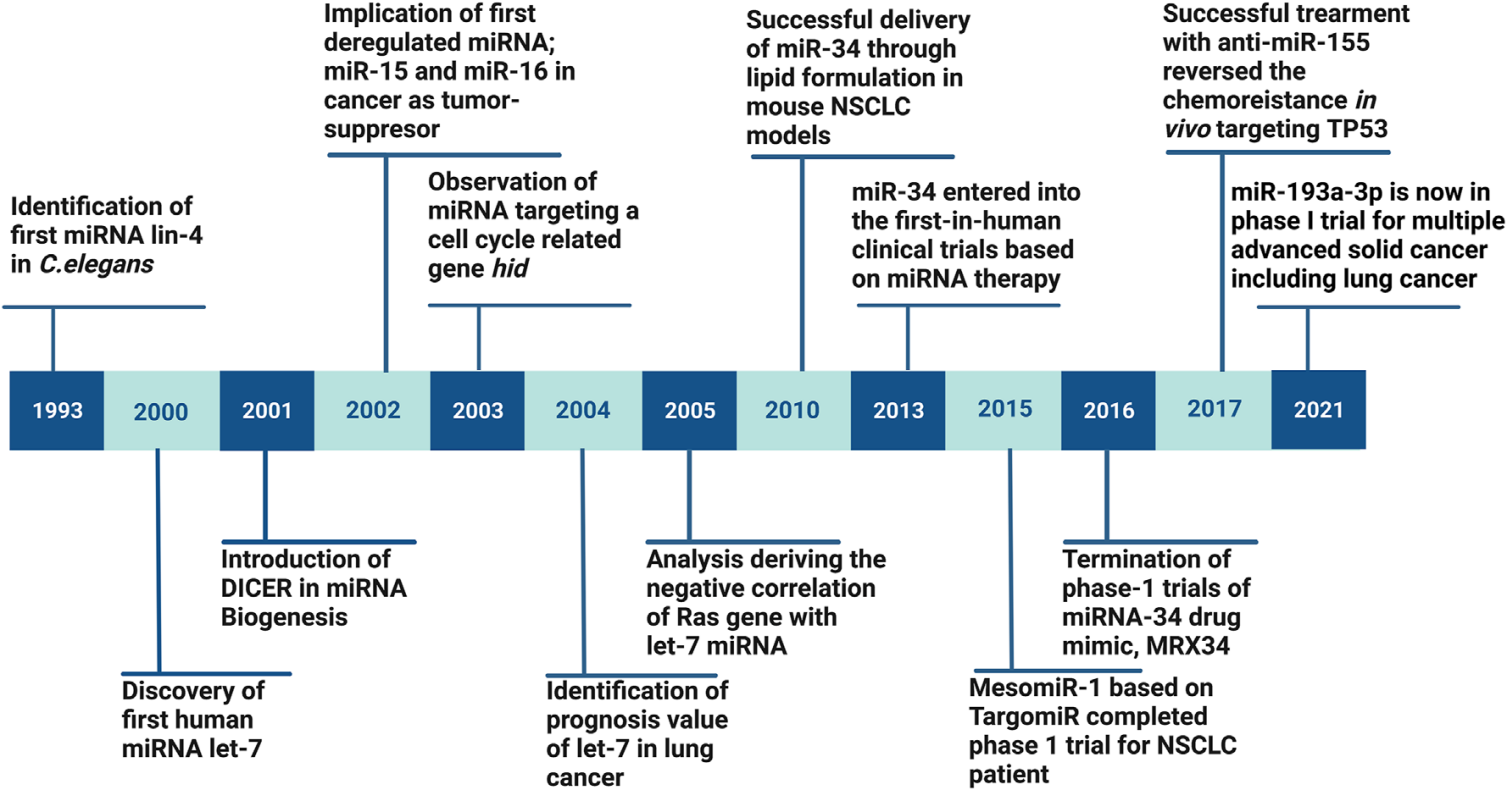
Timeline of miRNAs in Lung Cancer Research.

As of 10th March, 2022, 38,589 entries of miRNA have been recorded in miRBase-an online miRNA database dedicated to accumulating miRNA sequences and annotations [11]. However, the first miRNA ever to be reported was discovered nearly three decades ago from today [12, 13, 14]. In 1993, two independent but mutually shared studies in a singular issue of *Cell* reflected upon a novel regulatory mechanism by which it was concluded that *lin-4* did not actually encode a protein; rather corresponded to the complementary sequence of *lin-14* [12,13] (Figure 1). Thus, *lin-4* became the first such non-coding nucleotide sequence (later known as miRNA) [15] ever to have an impact on a major regulatory pathway in any known organism [16]. For 7 years, no other organism was identified with similar miRNA molecules until *let-*7 miRNA was discovered in *C. elegans* with a similar function as *lin-4* [17,18]. Multiple studies on different organisms corroborated the fact that *let-7* was present not only in nematodes but in plants, invertebrates and mammals as well [19, 20]. It was then for the first time the scientific committee concluded that miRNAs are evolutionarily conserved and a huge part of basic regulatory mechanisms in eukaryotes. Following a greater elaboration on the RNAi process (RNA interference) in 2000 [21] and introduction of DICER enzyme in miRNA biogenesis pathway in 2001 [22,23], miRNA 15 and miRNA 16 were documented to be the first miRNAs ever to be deregulated in cancer (B-cell lymphoma- Bcl2) [24,25]. In terms of miRNA's involvement in tumorigenesis, it was found in 2005 that miRNAs could also act as oncogenes now known as oncomiRs [26, 27]. On the other hand, a deep dive into the study of p53, the most common tumor suppressor gene, revealed that miRNAs could also function actively in the tumor suppressive mechanism [28, 29].

However, to reveal the mechanistic role of miRNAs in tumorigenesis, cell cycle regulation by miRNAs has to be disclosed first. Such involvement was confirmed by a study in 2003 on the *bantam* gene of drosophila revealing that the gene encoded a particular miRNA targeting an apoptotic gene *hid* which directly regulates apoptosis and cell cycle progression [30]. In 2006, the miRNAs miR-15a and miR-16-1 were reported to cause apoptosis by negatively regulating the anti-apoptotic *Bcl-2* gene in CLL cell lines [25]. miRNAs also share a long history with the pathogenesis of lung cancer in particular which has manifested them to be eligible candidates as biomarkers for the prognostic and diagnostic purposes [31]. In fact, the prognostic value of miRNA *let-7* was first elicited in lung cancer itself in 2004 as it represented a reduced expression in lung cancer patients leading to their poorer survival rate [32] (Figure 1). The next year, the first evidence ofmiRNA-target interaction was shown in a study on *C. elegans let-*7 miRNA targeting *let-60* encoding an ortholog of human oncogene RAS which in turn is deregulated in lung cancer as well [33]. Tumor suppressing roles of *let-7* were further explored in lung cancer; specifically in NSCLC (Non-Small Cell Lung Cancer) cells with a single nucleotide polymorphism (SNP) in K-RAS gene regulating the S-phase entry in cell cycle [34]. Furthermore, success from exogenous delivery of miR-34 through lipid formulation in mouse NSCLC models in 2010 [35] and following it up with another desired result next year-of miR-34a exerting apoptosis in NSCLC cell lines [36] led to the idea of developing potential therapeutics against lung cancer [37]. A transgenic approach to overexpress miR-17-92 cluster in lung cancer epithelial progenitor cells was also able to prevent tumor proliferation [38].

Following the determination of the prognostic value of *let-*7 miRNA in lung cancer [32], diagnostic value of the miRNAs was explored in lung cancer as well [16]. The stability of miRNAs in blood samples and tissue sections has been mentioned as one of the primary reasons behind choosing miRNAs as biomarkers and prognostic tools against lung cancer [39, 40, 41]. Excluding the possibility of non-specific side-effects from the miRNAs because of their complex molecular structure, miRNA delivery methods are still being examined and developed [42]. In 2013, miR-34a became the first miRNA ever to go into clinical trials (NCT01829971) for patients NSCLC or other solid and metastatic tumors [16] (Figure 1). To increase the efficacy of the miR-34a, combinatorial therapies have been suggested over the years in the form of miRNA-targeted drug [43], combination of different miRNAs [44] and combination of miRNA-siRNAs [45]. To avoid the off-target effects of the miRNAs, synthetic miRNA mimics are used which exert the same impact as the tumor suppressor miRNAs that were in the diseased tissues [46]. Most popularly, MRX34, the miR34a mimic encapsulated in lipid vesicle was developed by Marina Biotech Inc. [46]. and later went to clinical trials in 2014 [47] (Figure 1). As for the miRNAs that are up-regulated in different cancers, specific anti-sense oligonucleotides against them-now known as anti-miRNAs have been introduced [48]. The concept of antagomiR was first produced back in 2004 regarding the inhibition of the *let-*7 miRNA [49]. The very next year, the first mammalian trial with antagomiRs was conducted on mammals *in vivo* to silence multiple miRNAs through intravenous injection [50]. Another method of silencing oncomiRs involves locked nucleic acid (LNA) injection [51]. First report of LNA-anti-miRNA successfully making its mark was found in a 2008 study where the African Green Monkey showed a long-lasting and non-toxic resistance against miR-122 when inflicted with the corresponding LNA-anti-miRNA [52]. In terms of chemical drugs, Miravirsen became the first miRNA-inhibiting drug to ever enter clinical trial (SPC3649; NCT02031133) in 2010 [53]. In 2017, Katrien et al. investigated the function of miR-155 in chemotherapy resistance and evaluated anti-miR-155 treatment to reintroduce chemosensitivity. They have found that combination of anti-miR-155-DOPC with chemotherapy can be considered non-toxic and restored the drug induced apoptosis and cell cycle arrest in vivo [54]. Recently, Zhang et al. have disclosed that the discontinuation of MR34 occurred due to an effect called ‘TMTME’ (Too many targets for miRNA effect) [55]. In 2021, an LNP-formulated miR-193a-3pmimic developed by InteRNA Technologies has entered the phase I/Ib clinical trial. This phase I trial is still going on and the projected study completion date is December, 2023 [56].

Since the discovery of miRNA in roundworm, it has shown a great diversity from cancer formation to cancer treatment. However, to this date, no miRNA drug has been finalized as a drug for cancer.

## 3 Cell cycle and the role of miRNAs in cell cycle regulation

Cell cycle, the sequential process of cell division, is orchestrated by the highly regulated phases involving the activation or inactivation of positive and negative regulators. During the duplication process, genetic information of one cell generation is transmitted into the next one through the genome replication in S-phase and the segregation in M-phase. Two preparatory gaps are observed during this cyclic process where M is separated from S by the G1 phase and S is separated from M by the G2 phase. Hence, the timing, order, integrity and fidelity of cell cycle events are mainly monitored by a surveillance mechanism named cell cycle checkpoints. The checkpoints are triggered in G1 phase, S phase and during the G2/M transition in response to DNA damage and as a result cell growth is arrested to repair the damage. When the damage has been repaired, the cell cycle continues; otherwise, the cell is eliminated by apoptosis. The balance between cell proliferation and cell apoptosis is dependent on several families of proteins which are called key regulators of cell cycle showing specific functions in each phase, ultimately controlling the complex mechanism of cell division [57]. The cyclins-CDKs holoenzymes, and the E2F transcription factors play the role of positive regulator whereas the retinoblastoma protein pRB, and the cyclin-dependent kinases (CDK)-inhibitor families act as negative regulators in cell cycle [58]. Cyclins are proteins that control the transition between the phases by their action on the formation of a complex with cyclin-dependent kinases (CDKs). In addition, the catalytic activities of CDKs are dependent on the interactions with cyclins and Cdk inhibitors. However, an orderly manner of cell cycle progression is maintained by the closed coordination between this trio [59].

Different studies indicated that miRNAs are known to have an involvement in cell-cycle machinery by interacting with multiple cell cycle regulators through pairing to the 3′ untranslated region of target transcripts [60]. These non-coding miRNAs participate in cell cycle arrest by regulating the positive key regulators. For instance, miR-892b was found to target Cyclin D1 and Cyclin D2 which are the two key mediators of G1 phase [61]. miR-17-5p, a member of the miR-17-92 cluster, play a key role during G1/S cell cycle transition by targeting both cell proliferation inhibitor and activator such Cyclin D1, Cyclin D2, Cyclin G2, E2F3, E2F5, RB1 and p21 [62]. G1/S transition is modulated by miR-527 targeting Cyclin D1, p21, pRb, and Rb [63]. The cell cycle regulating factors cyclin E1, CDC25A, and Cyclin D1 have all been found to be directly targeted by miR-424 thus impair the G1/S or G2/M cell cycle transition in a variety of cell types [64]. The 3′-UTR of CDK6 was found to be targeted by miR-524-5p and thus arrest cell cycle in G1 phase [65]. Cyclin D1, a well-known cell cycle regulator, forms a complex with CDK4/6that promotes phosphorylation and deactivation of Rb in order to proceed from G1 to S phase. However, overexpression and accumulation of Cyclin D1 may function as oncogenic protein in several cancer cells and induce genetic changes in other regulatory proteins of cell cycle [66]. The expression of Cyclin D1 is reduced by the silencing effect of miR-20b and miRNA-373 [67,68].

E2Fs, a major transcription factor of cell cycle, apoptosis and differentiation, form complexes with the promoter of many diverse target genes which are associated with G1/S phase progression [69]. Two independent investigations revealed that miR-1258 and miR-30c could affect the expression of E2F1 and E2F7, respectively [70, 71]. The inhibitory role of miR-93 on G1/S-phase transition was conducted by antagonizing E2F1 and Cyclin D1, consequently inhibiting pRB/E2F1 pathway and several E2F1 downstream targets [72]. Although most of the cell-cycle-targeting miRNAs are responsible for cell cycle entry and the G1/S transition, a few studies found them to be involved in later phases of the cell cycle. After genome duplication, cell cycle control is mostly dependent on CDK1 in complex with Cyclin A or Cyclin B and the expression of these two cyclin proteins is downregulated by miR-125b, miR-24 and let-7 miRNAs [73, 74, 75].

miRNAs can also allow the cell to enter into the cycle and continue the progression by targeting negative regulators. For example, expression of the well-known tumor-suppressor, p53 has been regulated by miR-504 and miR-1285 [76]. Recent evidence suggests that miR-107 has been found to suppress the cell cycle transition at G1/S phase through inhibition of Cyclin E1 and subsequently effect on Rb phosphorylation at ser807/811 sites [77]. P21 is known as cyclin dependent kinase inhibitor and its action in the cell cycle is modulated by various miRNAs. p21 has been identified as a functional target of miR-6734, miR-512-5p, miR-1236, miR-208a, miR-146b, miR-33b-3p, miR-200c, indicating that miRNAs play a crucial role by modulating this cell cycle checkpoint regulator [78, 79, 80, 81, 82, 83, 84].

## 4 Involvement of deregulated oncomiRNA or tumor suppressor miRNA in lung cancer cell cycle

The cell cycle regulation is achieved by subsequent activation and inactivation of the oncogenes and tumor suppressors. miRNAs have been found to be directly regulated in such oncogenic and tumor suppressive networks [85, 86]. However, miRNAs that are overexpressed in tumor cells usually promote tumorigenesis as oncogenes or in this context, oncomiRs while tumor suppressor miRNAs exhibit the opposite effect (Table 1). OncomiRs normally inhibit the tumor suppressive activities by the genes controlling the cell apoptosis [37]. The entire miR-17-92 cluster including miR-17, miR-18a, miR-19a, miR-20a, miR-19b-1, miR-92-1 cluster has been declared as oncogenic by He et al. [87, 88]. The cluster has been reported to be upregulated in SCLC. Although, association with c-myc is the known regulatory mechanism for the miR-17-92 cluster to patronize the tumorigenesis process, inactivation of Rb has been additionally reported to be involved with the overexpression of the miRNA cluster in lung cancer screening [87]. Two more cases of SCLC oncomiRs directly modulating the core regulators were found- miR-25 was reported to cause SCLC tumorigenesis through regulating Cyclin- E2 [5] and miR-17-5p was reported regulating Rb12 [89]. miR-221-3p, miR-222-5p and miR-miR-194-5p increase survival rate of the NSCLC cells thus acting as oncogenic agents [90]. miR-205 has been noted as oncogenic in NSCLC tumorigenesis inhibiting p21 through SMAD4 downregulation [91]. Due to disruption in Drosha processing of miRNA precursors, some tumors result in a huge decline in mature miRNA levels [25]. miR-223, miR-186-3p and miR-186-5p have been found regulating CDK2 in suppressing lung adenocarcinoma [92] and NSCLC respectively [89]. MiR-613, miR-545 and miR-486-3p have been found regulating CDK4 in NSCLC suppression [89, 93, 94]. CDK6 was found to be regulated in multiple cases of tumor suppressing events between lung adenocarcinoma and NSCLC. All of the miRNAs- miR-137, miR-186-5p, miR-214-3p, miR-129-5p, miR-185, miR-107 and let-7 correlated with CDK6 expression in their respective tumor suppressive pathways [89, 95, 96, 97, 98]. MiR-138-5p has been found as the only miRNA regulating CDK8 in suppressing NSCLC [99]. In terms of Cyclins being regulated, Cyclin D1 was found to be regulated the most as miR-202, miR-206, miR-15, miR-9-5p, miR-15a, miR-16-5p, miR-193a-3p, miR-193b, miR-186, miR-545 and miR-146a-5p have been demonstrated impacting Cyclin D1 in their tumor suppression mechanism of lung cancers [7, 89, 93, 100, 101, 102, 103, 104].

**Table 1:** Dysfunction of OncomiRs and Tumor Suppressor miRNAs in the regulation of cell cycle causing lung cancer formation.

miRNA	Function of miRNA	Changes in Expression	Cell Cycle Regulators/Target Genes	Cancer types	References
miR-15a	Tumor Suppressor miRNA	Down-regulated	Cyclin D1, D2, E1	Non-small cell Lung Cancer	[101]
miR-16	Tumor Suppressor miRNA	Down-regulated	Cyclin D1, D2, E1	Non-small cell Lung Cancer	[101]
let-7	Tumor Suppressor miRNA	Down-regulated	c-Myc, CDK6	Lung Cancer	[98]
miR 107	Tumor Suppressor miRNA	Down-regulated	CDK6, Multiple Cyclins	Lung Cancer	[97]
miR 185	Tumor Suppressor miRNA	Down-regulated	CDK6, Multiple Cyclins	Lung Cancer	[97]
miR 186	Tumor Suppressor miRNA	Down-regulated	Cyclin D1, CDK2, CDK6	Non-small cell lung cancer	[103]
miR-34a ​and ​miR-15a/16	Tumor Suppressor miRNA	Down-regulated	Cyclin D1, CDK4, CDK6, E2F3	Non-small cell lung cancer	[110]
miR-25	Oncomir	Up-regulated	Cyclin E2, CDK2	Small-cell Lung Cancer	[5]
miR-202	Tumor Suppressor miRNA	Down-regulated	Cyclin D1	Lung Cancer	[100]
miR-145	Tumor Suppressor miRNA	Down-regulated	c-Myc	Non-small cell lung cancer	[111]
miR-613	Tumor Suppressor miRNA	Down-regulated	CDK4	Non-small cell lung cancer	[112]
miR-138	Tumor Suppressor miRNA	Down-regulated	Cyclin D3	Non-small cell Lung Cancer	[113]
miR-17	Oncomir	Up-regulated	Rb	Non-small cell Lung Cancer	[87]
miR-18a	Oncomir	Up-regulated	Rb	Non-small cell Lung Cancer	[87]
miR-19a	Oncomir	Up-regulated	Rb	Non-small cell Lung Cancer	[87]
miR-20a	Oncomir	Up-regulated	Rb	Non-small cell Lung Cancer	[87]
miR-19b-1	Oncomir	Up-regulated	Rb	Non-small cell Lung Cancer	[87]
miR-205	Oncomir	Up-regulated	p21	Non-small cell Lung Cancer	[87]
miR-30d-5p	Tumor Suppressor miRNA	Down-regulated	Cyclin E2	Non-small cell Lung Cancer	[106]
miR-545	Tumor Suppressor miRNA	Down-regulated	Cyclin D1, CDK4	Lung Cancer	[93]
miR-17-5p	Oncomir	Up-regulated	Rb12	Small-cell Lung Cancer	[89]
miR-221-3p	Oncomir	Up-regulated	p27	Non-small cell Lung Cancer	[90]
miR-222-5p	Oncomir	Up-regulated	p27	Non-small cell Lung Cancer	[90]
miR-194-5p	Oncomir	Up-regulated	p27	Non-small cell Lung Cancer	[90]
miR-223	Tumor Suppressor miRNA	Down-regulated	CDK2	Lung Cancer	[92]
miR-129-5p	Tumor Suppressor miRNA	Down-regulated	CDK6	Lung Cancer	[96]
miR-214-3p	Tumor Suppressor miRNA	Down-regulated	CDK6	Lung Cancer	[89]
miR-137	Tumor Suppressor miRNA	Down-regulated	CDK6	Lung Cancer	[95]
miR-186-5p	Tumor Suppressor miRNA	Down-regulated	CDK2, CDK6	Non-small cell Lung Cancer	[89]
miR-206	Tumor Suppressor miRNA	Down-regulated	Cyclin D1	Non-small cell Lung Cancer	[89]
miR-15	Tumor Suppressor miRNA	Down-regulated	Cyclin D1	Non-small cell Lung Cancer	[89]
miR-9-5p	Tumor Suppressor miRNA	Down-regulated	Cyclin D1	Non-small cell Lung Cancer	[89]
miR-16-5p	Tumor Suppressor miRNA	Down-regulated	Cyclin D1	Non-small cell Lung Cancer	[89]
miR-193a-3p	Tumor Suppressor miRNA	Down-regulated	Cyclin D1	Non-small cell Lung Cancer	[7]
miR-193b	Tumor Suppressor miRNA	Down-regulated	Cyclin D1	Non-small cell Lung Cancer	[102]
miR-34 Family	Tumor Suppressor miRNA	Down-regulated	p53	Non-small cell Lung Cancer	[107]
miR-340	Tumor Suppressor miRNA	Down-regulated	p27	Non-small cell Lung Cancer	[109]
miR-146a-5p	Tumor Suppressor miRNA	Down-regulated	Cyclin D1, Cyclin D2	Non-small cell Lung Cancer	[104]
miR-486-3p	Tumor Suppressor miRNA	Down-regulated	CDK4	Non-small cell Lung Cancer	[6]
miR-15-5p	Tumor Suppressor miRNA	Down-regulated	Cyclin D, Cyclin E	Non-small cell Lung Cancer	[105]
miR-16-5p	Tumor Suppressor miRNA	Down-regulated	Cyclin D, Cyclin E	Non-small cell Lung Cancer	[105]
miR-138-5p	Tumor Suppressor miRNA	Down-regulated	CDK8	Non-small cell Lung Cancer	[99]
miR-197	Oncomir	Up-regulated	p53	Non-small cell Lung Cancer	[114]
miR-34b-3p	Tumor Suppressor	Down-regulated	CDK4	Non-small cell Lung Cancer	[115]
miR-144	Tumor Suppressor	Downregulated	Cyclin E1/E2	Non-small cell Lung Cancer	[116]
miR-224	OncomiR	Up-regulated	p21	Lung Adenocrcinoma	[117]
miR-330	Tumor Suppressor	Down-regulated	E2F1	NSCLC	[118]
miR-143	Tumor suppressor	Down-regulated	CDK1 and CDK4	Lung cancer	[119]
miR-506	Tumor-suppressor	Down-regulated	CDK1 and CDK4	Lung cancer	[119]
miR-433	Tumor-suppressor	Down-regulated	E2F3	NSCLC	[120]
miR-150	OncomiR	Up-regulated	p53	NSCLC	[121]
miR-122-5p	OncomiR	Up-regulated	p53	NSCLC	[122]
miR-3607-3p	Tumor Suppressor	Down-regulated	Cyclin E2	NSCLC	[123]
miR-208a	OncomiR	Up-regulated	p21 (nuclear)	NSCLC	[81]
miR-708-5p	Tumor suppressor	Down-regulated	p21 (cytoplasmic)	Lung cancer	[124]
miR-155	OncomiR	Up-regulated	p53	Lung cancer	[54]

**Figure 2:**
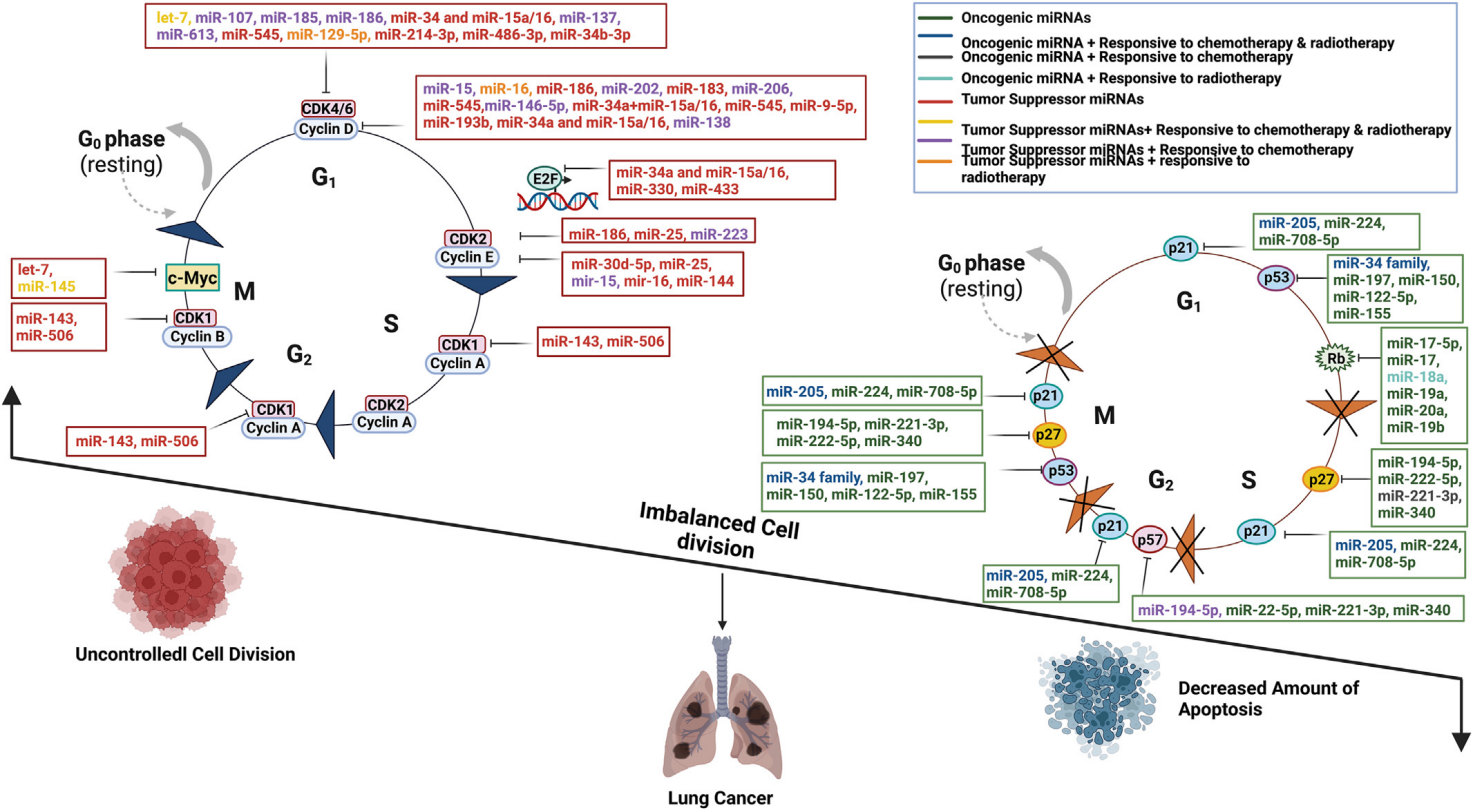
The Role of miRNA in lung cancer formation.

Among them, miR-146a-5p and miR-15a have been found regulating Cyclin D2 as well [101, 104]. miR-15-5p, miR-16-5p and miR-30d-5p were found significantly active in tumor suppression of NSCLC through regulation of Cyclin E [105, 106]. Multiple other cyclins were reported to be involved in these suppression mechanisms of lung cancer regulated by miR-107 and miR-185 [97]. As for the negative regulators, p53-the most popular tumor suppressor was regarded popular in this case again as the miR-34 family was shown to be tumor suppressors of differential lung cancers through p53 regulation [107]. A positive feedback loop constructed by p53 and miR-34a particularly was discovered to be an effective tumor suppressive machinery in lung cancer tumorigenesis [108]. Other miRNAs that had to activate p53 to suppress NSCLC were found to be-miR-221-3p, miR-222-5p and miR-194-5p [90]. Another negative regulator from the cip/kip family-p27 was invoked to cause cell cycle arrest in NSCLC by the tumor suppressor miR-340 [109]. Apoptosis by Rb was caused by miR-15a, miR-16 and miR-34a [110]. The imbalance between the cell cycle progression and cell apoptosis has occurred due to the deregulated expression of oncomiR and Tumor suppressor miRNA which subsequently lead to lung cancer formation (Figure 2).

## 5 Cell cycle associated miRNAs in lung cancer treatment

### 5.1 miRNA as biomarker

miRNAs have been deduced as a powerful set of determinants as prognostic or diagnostic biomarkers or predictors of diseases to design a therapeutic strategy accordingly [125, 126]. This decision has been constantly driven forward upon reflecting on the diagnostic and prognostic utility of the miRNAs [26] for the first time through the establishment of *let-*7 miRNA as a prognostic tool against lung cancer [32]. Within a contemporary period, the idea of employing miRNA signatures against diseases only strengthened the idea for miRNAs to be considered as biomarkers in diseased tissues. Also, miRNA expression profile based cancer detection techniques across differential developmental stages were recognized [127].

Naturally, miRNA expression pattern is uniquely variable in specified tissues and cells. Therefore, miRNAs can be expressed differentially and distinctly in numbers in tumor tissues as opposed to the normal tissues in a particular cancer case [128]. On top of that, depending on the subsequent stages of cancer, the expression of the miRNAs may change significantly upholding a significant view of correlation between the tumorigenesis of the case, metastasis in specific tissues and the corresponding signature miRNA expression [129]. Such clinical relevance of the biomarker miRNAs in cancer tissues has been brought to attention through selection of specific unique sequences of miRNAs in both the cancer tissues and the corresponding normal tissues and more conveniently through analysis of the expression profile of miRNAs after biopsies or surgeries to remove the tumor tissues.

In case of lung cancer in particular, there have been multiple studies focusing solely on the miRNA deregulation in prognostic, diagnostic and predictive approaches towards treatment [130, 131, 132]. Considerable changes in concentrations and over or under-expressions of the miRNAs under tumor conditions have rallied them as suitable biomarkers for lung cancer [133, 134]. More condensed efforts into NSCLC rather than other forms of lung cancer have revealed how miRNAs are potentially justified in terms of early diagnosis and prognosis of NSCLC [135, 136]. One of the reasons behind miRNAs acting differently with differential levels of expressions in tumor and corresponding normal cells is them being deregulated by cell cycle proteins. Therefore, instead of a generalized amalgamation of miRNAs as biomarkers against lung cancer, we attempted to investigate only those miRNAs targeting our listed core regulators as delineated in Table 2.

**Table 2:** MiRNAs as diagnostic and prognostic biomarkers in lung cancer.

miRNA	Outcome of patients	Cancer type	Sample size/type	Statistical method	Reference
miR-15a	Diagnosis (P < 0.05)	Non-small cell Lung Cancer	17 patients/tissue	Student's *t* test	[139]
let-7	Lower survival (p < 0.05)	Non-small cell lung cancer	31 patients/tissue	Pearson's correlation analysis	[142]
miR-107	Poor OS (p = 0.007)	Non-small cell lung cancer	137 patients/tissue	Kaplan-Meier analysis with the log-rank test	[145]
miR-185	Poor OS (p = 0.004)	Non-small cell lung cancer	146 patients/serum	Kaplan-Meier	[141]
	Lower RFS (p = 0.036)				
miR-186	Potential diagnosis P < 0.001	Non-small cell lung cancer	62 patients/serum Exhaled Breath Condensate	ROC curve analysis	[138]
miR-25	Shorter OS (p = 0.046) and RFS(p = 0.0362)	Non-small cell lung cancer	128 patients/serum	Kaplan-Meier analysis with the log-rank test	[140]
miR-145	Shorter DFS (p = 0.007)	Non-small cell lung cancer	48 patients/tissue	Kaplan-Meier analysis	[146]
miR-17-5p	Shorter OS (p = 0.035)	Non-small cell lung cancer	221 patients/serum	Cox proportional hazards regression model	[158]
miR-18a	Worse DFS (p < 0.001) and Shorter OS (p < 0.001)	Non-small cell lung cancer	196 patients/serum	Kaplan-Meier analysis with the log-rank test	[148]
miR-19b	Decreased OS (p = 0.003)	Non-small cell lung cancer	61 patients/tissue	Kaplan-Meier analysis	[143]
miR-16-5p	Diagnosis (p < 0.0001)	Non-small cell lung cancer	94 patients/serum	ROC curve analysis	[137]
miR-340	Lower OS (p < 0.05) and DFS (p < 0.05)	Non-small cell lung cancer	64 patients/tissue	Kaplan-Meier analysis	[144]
miR-146a	Worse OS (p = 0.002)	Non-small cell lung cancer	66 patients/serum	Kaplan-Meier analysis	[143]
miR-197	Worse OS	Non-small cell lung cancer	124 patients/tissue	Kaplan-Meier analysis	[150]
miR-154	Lower OS (P < 0.001)	Non-small cell lung cancer	40 patients/tissue	Kaplan-Meier analysis	[149]
miR-3607-3p	Shorter Survival (p < 0.01); Diagnosis (p < 0.01)	Non-small cell lung cancer	162 patients/tissue; 80 patients/serum	Kaplan-Meier analysis; ROC curve analysis	[123]

Numerous studies have significantly demonstrated that miRNA might be an effective screening factor either in diagnostic or prognostic implications against NSCLC.

Over the years, serum miRNAs have been established as formidable biomarkers for early detection of NSCLC. From out of our specifically categorized miRNAs, serum miR-16-5p was reported to be an early diagnostic factor for NSCLC in a 2016 study [137]. miR-186 found in both serum and exhaled breath condensate also acts as a diagnostic biomarker for NSCLC detection [138]. As for tissue miRNAs, miR-15a expression is independently and significantly changed in NSCLC and so confirmed to be a probable diagnostic biomarker [139]. miR-3607-3p has been solidly propounded as a novel diagnostic biomarker when collected from serum. However, the same miRNA from tissue samples corresponds to the shorter survival analysis with p < 0.01 implying its prognostic value as a biomarker as well [123]. Such a dual role of both diagnostic and prognostic nature for NSCLC is also attributed to serum miR-25 as seen from Li et al. [140] and serum miR-185 in NSCLC samples as seen from Liu et al. [141]. In terms of pure prognostic factors, let-7 was one of the first ones to have a predictive correlation with lung cancer and Xia et al. showed how lower expression of let-7 along with higher K-Ras expression was indicative of prognosis of NSCLC [142]. Wu et al. released their work on two miRNAs- miR-19b and miR-146a with contrasting serum levels for NSCLC prediction and termed both as prognostic biomarkers for NSCLC survival [143]. Lowering the expression of miR-340 in NSCLC tissues results in poor prognosis and so Qin et al. experimented by overexpressing miR-340 which targets the cell cycle regulator CDK4 to check tumor cell proliferation [144]. Zhong et al. established miR-107 from tissue samples as a novel prognostic marker since it represented decreased expression in NSCLC which was significantly associated with tumorigenesis and shorter survival in patients [145]. Tissue miR-145 also correlates with poor prognosis of NSCLC when under-expressed [146]. However, there may be instances where significantly higher expression of miRNAs in tumor cells deems them to be prognostic factors as well. In a study with 221 NSCLC patients in the mix, high level of serum miR-17-5p pertained to shorter survival in patients whereas a lower expression extended their survival [147]. Similarly, miR-18a being an oncogenic miRNA accounts for shorter overall survival in various cancers including NSCLC when overexpressed-making it one of the independent prognostic markers in the treatment scene [148]. It can be noticed how frequently shorter overall survival statistics is used in estimating patient's prognosis just as in the case of two tissue miRNAs in our list-miR-154 and miR-197- both of which have been suggested as novel biomarkers for NSCLC in terms of prognosis of the cancer [149, 150]. miR-200 family, especially its constituent members-miR-200a, miR-200b, miR-200c and miR-141 have been found to be playing significant roles as prognostic or diagnostic biomarkers in lung cancer as well. However, their roles are verily inconsistent as they're expressed in contrasting concentration in different lung cancer subtypes regardless of samples-tissues or serum [151, 152]. For example, miR-200b seems to inhibit tumor progression of lung cancer [153]but is overexpressed with shorter survival analysis in NSCLC patients [154]. miR-200c also displays a similar tumor suppressing property in lung cancer [155] but is overexpressed in NSCLC [156]. On top of that, despite a report of miR-200b targeting E2F3 to abrogate chemoresistance, no conclusive remarks have yet been reached in terms of directly relating miR-200 family to the core cell cycle regulators as biomarkers in lung cancer [157]. Hence, we had to omit any member from miR-200 family from entering our list of eligible prognostic or diagnostic miRNA biomarkers associated with core cell cycle regulators.

### 5.2 miRNAs in lung cancer therapy

**Figure 3:**
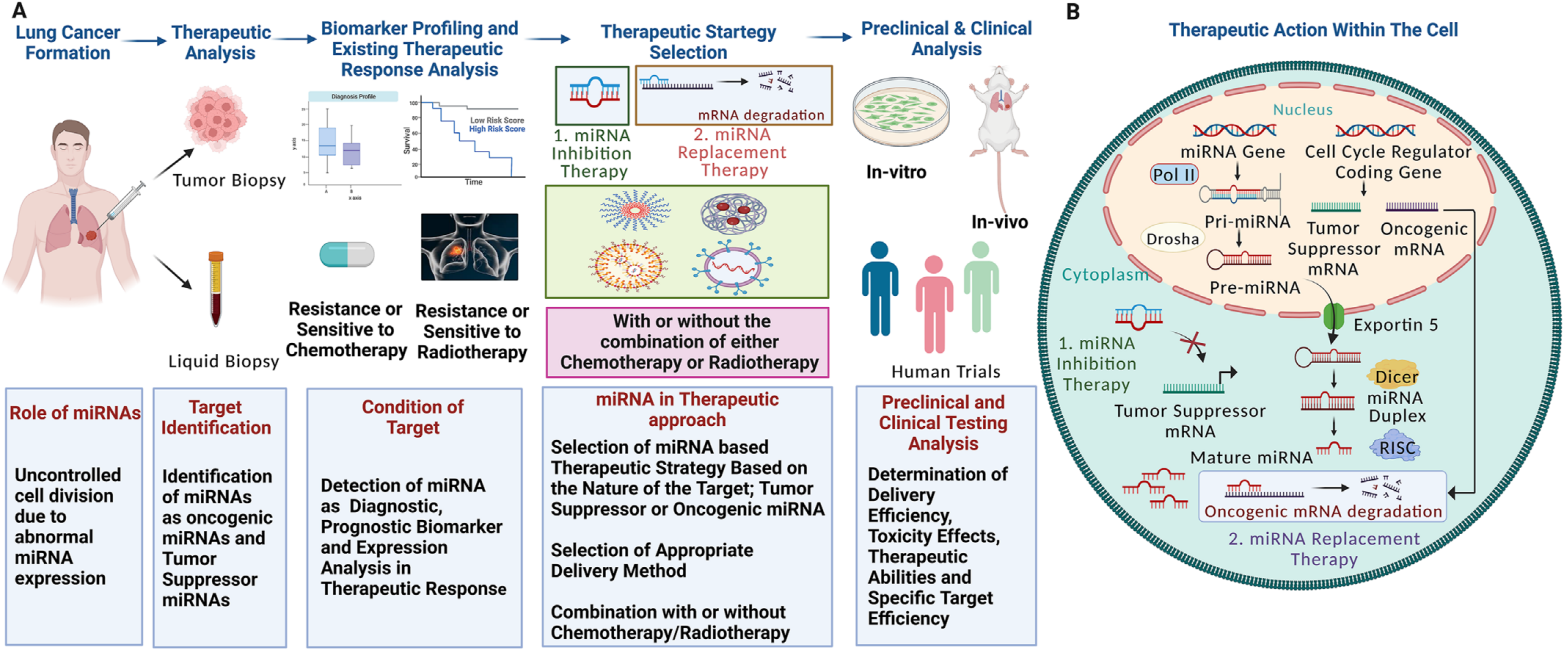
(a) Therapeutic potentiality of miRNA in lung cancer treatment from target identification to treatment approach. (b) Diagrammatic representation of therapeutic mode of action of miRNAs within the cell structure during miRNA inhibition therapy and miRNA replacement therapy.

As miRNAs have been reported to interact with cell cycle regulation by targeting a wide range of cell cycle associated gene and as well as their ability to restore balanced gene network, they have become an ideal target for human cancer treatment. However, the potential to use miRNAs as diagnostic and prognostic tools has helped in the understanding of miRNA mediated pathogenesis in cancer, which in turn aided in making appropriate therapeutic decisions throughout the treatment process (Figure 3a). In comparison to protein-based therapeutic molecules and even plasmid DNA-based gene therapy, miRNAs have shown less toxicity and decreased immune response as they contain natural antisense nucleotides. Despite the fact that ribonuclease may easily cleave the unprotected 3′-hydroxyl and 5′-phosphate ends of miRNAs, resulting in transitory expression and a limited half-life, this stability issue can be overcome by employing Argonaute 2 protein or naturally present extracellular vesicles [159]. However, miRNA has emerged as a vast research landscape for academic and clinical research laboratories, pharmaceutical industries, and biotech companies. In case of lung cancer therapy, miRNAs are being applied in two main strategies, either restoring the function of tumor-suppressor miRNA or inhibiting the action of oncomiRNAs (Figure 3b).

#### 5.2.1 Restoration therapy of miRNA

Tumor suppressor miRNAs have been found to be lost or downregulated in lung cancer cells and in that case synthetic miRNA mimics or viral vectors expressing specific miRNAs can be applied to induce apoptosis and decrease tumor growth by restoring the function of tumor suppressor miRNAs. As miRNA mimics are small and can easily be modified, they have drawn much attention *in vitro* and *in vivo* models. After the successful transfection of miRNA mimic into the cell, they act as mature endogenous miRNA that bind with the target gene to repress translational expression. In addition, transfection of miR-340 mimic into NSCLC cells has reported to reduce cell proliferation and induce apoptosis by silencing multiple key negative regulator of p27 [160]. For maintaining the increased amount of tumor suppressor miRNAs, another strategy of restoration therapy can be used where cancer cells are transformed with vectors such as lentiviral, adenoviral, and retroviral expressing deficient miRNAs. For example, *let-7* containing adenoviral and lentiviral delivery showed significant result in reducing colony formation of lung tumor in a KRAS driven mouse model [161, 162].

#### 5.2.2 Inhibition therapy of miRNA

Another approach to reduce the progression of cancer cell is inhibiting the function of oncogenic miRNAs applying antagomiR, locked nucleic acid (LNA) miRNAs and miRNA sponges. AntagomiRs play an opposite role by preventing the function of oncomiR from binding to the 3′-UTR of mRNA, allowing the mRNA to be translated [163]. Due to lack of immune response activation, antagomiR can be an essential aspect for therapeutic use whereas large dosages requirement for its effective miRNA blocking action may limit its clinical use. In that case, multiple small dosages of antagomiR drug can be applied over a period of time to treat lung cancer.

Locked Nucleic Acid (LNA) is an RNA derivative that has recently received great attention as an effective therapeutic option for modulating miRNA expression. In LNA, the methylene bridge forms a bi-cyclic structure connecting the 2′-oxygen atom and the 4′-carbon atom of the ribose ring to lock the ribose which makes it highly stable and less toxic [164]. Additionally, Miravirsen, an LNA-based therapeutic has already completed a phase II clinical trial targeting miR-122 in chronic hepatitis C patients [165] and it may serve as a guide to improve the delivery of LNAs to lung cancer cells to establish the possibility of this novel therapeutic approach in the fight against lung cancer. A recent *in vivo* and *in vitro* analysis has already shown that neutralization of oncogenic miR-197 as well as the up-regulation of p53 can be done using LNA-anti-miR-197 in lung cancer [166]. Another important strategy to inhibit the activity of oncogenic miRNAs is introducing miRNA sponges which have specific binding sites for the miRNA seed region permitting them to block a large family of related miRNAs. When the vectors containing this competitive inhibitor are transfected into the cells, it binds to the oncogenic miRNA and makes the target mRNA free to transcribe [167]. In a research study, transfection of miR-26a-5p sponge in mouse xenograft model revealed that it can suppress tumorigenesis in NSCLC by silencing the over-expressed miR-26a-5p [168].

## 6 Combination of miRNA with traditional lung cancer therapy

### 6.1 miRNAs in lung cancer radiotherapy

Radiotherapy has emerged as a popular form of treatment against multiple tumors with distinctive therapeutic success so far [169]. The basic principle of radiotherapy is producing free radicals in neoplastic cells-mostly on DNA by effecting destruction of tumors by ionizing radiation [170]. Improvement in radiotherapy has been achieved by combinatorial addition of chemotherapy, targeted therapy or any other types of therapies including surgeries [171]. Cases of many tumors developing radio resistance are already being observed now leaving a huge question mark on the accuracy of radiotherapy. So, to successfully optimize radiotherapy against any tumor, radiosensitivity, as opposed to the radio resistance, of the tumor has to be increased-as in maximizing the number of tumor cells killed while retaining a good number of normal cells and minimizing side-effects [172, 173].

Radiotherapy basically triggers DDR (DNA Damage Response) which in turn opts either for DNA damage repair or instant apoptosis based on the tumor condition [174]. miRNAs on the other hand have been found working through the DDR mechanism itself at the post-transcriptional level by regulating specialized targets expression [175, 176]. So, DNA damage response to radiation is evidently mediated by miRNAs leading to the fact that radio-sensitivity can be controlled by regulating specific miRNAs [177].

The most popular specimen data analytical studies on LC lines evaluating miRNA regulation against radiotherapy include Wang et al. finding 12 differentially expressed miRNAs in 30 radiosensitive and radioresistant NSCLC specimens and succeeding to cause an increase in radio-sensitivity by overexpressing miR-126 [9] and Chen et al. finding 14 miRNAs correlated with radioresistant genes and 5 with radiosensitive ones and concluding with a 4 miRNA signature showing higher expression in responders [178]. These studies emphasize the fact that miRNAs are involved in the resistance and sensitivity towards the radiotherapy treatment in lung cancer (Figure 1).

In our study, we have taken a rather literature-based approach coherent with the rest of our review, in which we attempted to extract information only on our shortlisted miRNAs corresponding to the key cell cycle regulators in lung cancer. 12 specimen data analysis-based studies on lung cancer were skimmed through to identify 11 miRNAs (miR-34a, miR-18a, miR-16-5p, let-7, miR-138, miR-25, miR-145, miR-205, miR-545, miR-129-5p and miR-206) from our initial list that have been reported to be altering radio-sensitivity in clinical favor by either being overexpressed or downregulated (Table 3). However, miRNA mimics have been used for 7 miRNAs (miR-34a, let-7, miR-16-5p, miR-138, miR-145, miR-205 and miR-129-5p) and were found to be overexpressed to increase radio-sensitivity through a variety of mechanisms (Table 3) [179, 180, 181, 182, 183, 184, 185, 186]. Two separate studies were noted on miR-34a altering radiosensitivity in NSCLC- one of them reporting its involvement in regulation of RAD51 gene and the other in suppression of LyGDI signaling pathway [179, 180]. Yang et al. detailed how miR-138 overexpression suppresses radio sensitization-related gene SENP1 to facilitate radio sensitization of lung cancer cells and how changes in cell cycle accompany the process [183]. A recent study has suggested miR-145 to be a novel radiosensitizer of NSLC as it suppresses the TMOD3 at protein levels [184]. miR-205 mimics have been therapeutically successful in sensitizing tumor in xenograft models by inhibiting ZEB1 and Ubc13 and miR-129-5p mimic have found to significantly increase radiosensitivity in NSCLC by targeting SOX4 and RUNX1 [185,186]. On the flip side, depleting miR-18a by antagomir resulted in increased radio-sensitivity in NSCLC [187]. He et al. had proved miR-25 overexpression regulating radiotherapy sensitivity in NSCLC by inhibiting BTG2 expression-indicating towards an antagomiR-based therapy against the miRNA for achieving sensitization to radiotherapy [188]. Contrarily, two antagomiR-based studies against miR-545 and miR-206, revealed that the antagomiR action in downregulating the miRNAs rather decreased the radio-sensitivity in samples [185, 189]; indirectly concluding that the clinical overexpression of these miRNAs through mimic input may therapeutically increase radio-sensitivity in the tumors.

**Table 3:** miRNAs associated with sensitivity to Lung Cancer Radiotherapy.

Target miRNA	Cancer	miRNA Regulation System	Expression Level of miRNA	Cell Lines	Response Observed	References
miR-34a	NSCLC	mimic	Overexpression	A549 and H1299	Radiosensitivity ↑	[179]
miR-34a	NSCLC	mimic	Overexpression	A549, H1299, H460, and Calu6	Radiosensitivity ↑	[180]
miR-16-5p	NSCLC	mimic	Overexpression	GLC-82 and HTB-182	Radiosensitivity ↑	[181]
let-7	NSCLC	mimic	Overexpression	A549/IR	Radiosensitivity ↑	[182]
miR-138	Lung Adenocarcinoma	mimic	Overexpression	A549	Radiosensitivity ↑	[183]
miR-25	NCLC	antagomiR	Downregulation	H226, H1299, A549 and U1810	Radiosensitivity ↑	[188]
miR-145	NSCLC	mimic	Overexpression	A549 and H460	Radiosensitivity ↑	[184]
miR-18a	NSCLC	antagomiR	Downregulation	A549	Radiosensitivity ↑	[187]
miR-205	NSCLC	mimic	Overexpression	A549	Radiosensitivity ↑	[185]
miR-545	Lung Carcinoma	antagomiR	Downregulation	C57-BL/6	Radiosensitivity ↓	[189]
miR-129-5p	NSCLC	mimic	Overexpression	A549 and H1299	Radiosensitivity ↑	[186]
miR-206	NSCLC	antagomiR	Downregulation	H1650, H460 and A549	Radiosensitivity ↓	[190]

### 6.2 miRNAs in lung cancer chemotherapy

A widely-practiced therapy option for cancer treatment is chemotherapy which offers a massive scope of therapeutics in lung cancer as well. However, development of multiple drug resistance (MDR) has been a major issue in reversing the chemotherapy effects as the tumor cells show resistance to numerous drugs at a time [191]. Sometimes, cancer cells, as seen in NSCLC lines, may escape apoptosis triggered by chemotherapeutic drugs by simply under-expressing pro-apoptotic proteins or overexpressing anti-apoptotic ones [192] or inducing autophagy within [193]. Along with the obvious finding of being differentially expressed in tumor tissues miRNAs have also been evidently shown to be corresponding with drug response (Figure 1). In case of NSCLC cell lines, different members of the ABC family may be regulated by different miRNAs and may condition chemoresistance or chemosensitivity [194]. For example, chemoresistance in lung cancer can be grown through modulating the p53 cell cycle regulator family led by a single miRNA as reported by Hong et al. [10]. Contrarily, miRNAs like miR-34a and others have been predicted to increase chemosensitivity as well in NSCLC [195, 196].

In our study, we aimed to take only the shortlisted miRNAs into consideration as we looked to figure out their roles in chemosensitivity or chemoresistance against Lung Cancer. We finally jotted down 23 studies compiling researches on 19 miRNAs from our list that provided with conclusive remarks as to whether they were expressed in favor of chemosensitivity in therapeutic approaches against Lung cancer or not (Table 4). 14 out of those 23 studies were condensed on the drug Cisplatin. miRNA mimics were used in case of 16 miRNAs (miR-34a, miR-15a, let-7, miR-107, miR-138, miR-185, miR-186, miR-202, miR-145, miR-613, miR-221-3p, miR-194-5p, miR-137, miR-206, miR-146a and miR-146a-5p) as all of the miRNAs were concluded to be overexpressed in sensitizing lung cancer samples to a stretch of chemotherapeutic drugs. MiR-145 was found to have the most studies done on in chemotherapy effects. Three of those studies directly mimicked its effect in tumor cells against Gefitinib [197], Erlotinib [198]and Pemetrexed whereas the other showed how sponging miR-145 by a specific lncRNA resulted in decreased chemosensitivity to Cisplatin in NSCLC [199]. miR-185, miR-145 and miR-186-5p directly target the ABC transporter family in their sensitization process to a number of chemotherapeutic drugs [199, 200, 201]. Song et al. reports miR-34a sensitizing lung cancer cells to Cisplatin through the cell cycle inhibitor p53 regulation on MYCN axis [202]. miR-15a was found elevating the anti-cancer effect of Cisplatin in NSCLC as well in a 2016 study [203]. A similar study was carried out by Sun et al. in case of miR-202 as it displayed an anti-tumor effect as well against NSCLC by sensitizing them to Cisplatin [204]. Tumor suppressive role of overexpressed miR-613 was explored by Luo et al. as they concluded for the mimic application of the miRNA for sensitizing NSCLC cells to Cisplatin [205]. miR-137 mimics have succeeded in sensitizing lung cancer cell lines to both Cisplatin and Paclitaxel [206]. Other than Cisplatin, Paclitaxel sensitivity could be enforced in NSCLC by mimicking miR-107 and miR-221 which target Bcl-wand MDM2, respectively [207, 208]. Moreover, miR-194-5p increases chemosensitivity to Doxorubicin in NSCLC cell lines [209]. Adriamycin is another drug that is listed which NSCLC has been found sensitized to when mimicking miR-138 [210]. As for picking antagomiRs, anti-miR-205, anti-miR-223 and anti-miR-15-5p resulted in sensitization of NSCLC to Cisplatin [211], Doxorubicin and Cisplatin [212, 213] and Nobiletin [214], respectively. However, anti-miR-138-5p rather showed desensitization of NSCLC to Cisplatin implying that overexpression of miR-138-5p would retain chemosensitivity in NSCLC against the Platinum drug [215].

**Table 4:** miRNAs associated with sensitivity to Lung Cancer Chemotherapy.

Target miRNA	Cancer	Regulation System	Expression level	Cell Lines	Corresponding Drug	Response Observed	References
miR-34a	NSCLC	mimic	Overexpression	H226, H1299, A549, H1975 and HCC827	Cisplatin	Chemosensitivity ↑	[202]
miR-15a	NSCLC	mimic	Overexpression	Calu1	Cisplatin	Chemosensitivity ↑	[203]
let-7	NSCLC	mimic	Overexpression	A549	Cisplatin	Chemosensitivity ↑	[182]
miR-107	NSCLC	mimic	Overexpression	A549	Paclitaxel	Chemosensitivity ↑	[207]
miR-138	NSCLC	mimic	Overexpression	A549 and NCI–H23	ADM-based chemotherapeutics	Chemosensitivity ↑	[210]
miR-185	NSCLC	mimic	Overexpression	A549	Cisplatin	Chemosensitivity ↑	[200]
miR-186	NSCLC	mimic	Overexpression	A549 and H1299	Paclitaxel	Chemosensitivity ↑	[201]
miR-202	NSCLC	mimic	Overexpression	A549 and NCI–H441	Cisplatin	Chemosensitivity ↑	[204]
miR-145	NSCLC	mimic	Overexpression	PC-9	Gefitinib	Chemosensitivity ↑	[197]
miR-145	NSCLC	mimic	Overexpression	A549	Erlotinib	Chemosensitivity ↑	[198]
miR-145	LUAD	mimic	Overexpression	A549	Cisplatin and Pemetrexed	Chemosensitivity ↑	[199]
miR-613	NSCLC	mimic	Overexpression	A549 and H1299	Cisplatin	Chemosensitivity ↑	[205]
miR-205	NSCLC	AntagomiR	Downregulation	A549	Cisplatin	Chemosensitivity ↑	[211]
miR-221-3p	NSCLC	mimic	Overexpression	A549	Paclitaxel	Chemosensitivity ↑	[208]
miR-194-5p	NSCLC	mimic	Overexpression	A549	Doxorubicin	Chemosensitivity ↑	[209]
miR-223	NSCLC	AntagomiR	Downregulation	A549, NCI–H358, NCI–H1299 and HCC827	Doxorubicin	Chemosensitivity ↑	[212]
miR-223	NSCLC	AntagomiR	Downregulation	A549, NCI–H358, and NCI–H1299	Cisplatin	Chemosensitivity ↑	[213]
miR-137	NSCLC	mimic	Overexpression	A549	Cisplatin and Paclitaxel	Chemosensitivity ↑	[206]
miR-206	LUAD	mimic	Overexpression	A549 and H1299	Cisplatin	Chemosensitivity ↑	[216]
miR-146a	NSCLC	mimic	Overexpression	A549 and SPC-A1	Cisplatin	Chemosensitivity ↑	[217]
miR-146a-5p	NSCLC	mimic	Overexpression	A549	Cisplatin	Chemosensitivity ↑	[218]
miR-15-5p	NSCLC	AntagomiR	Downregulation	A549 and H460	Nobiletin	Chemosensitivity ↑	[214]
miR-138-5p	NSCLC	AntagomiR	Downregulation	A549	Cisplatin	Chemosensitivity ↓	[215]

### 6.3 miRNAs in lung cancer immunotherapy

Tumors must weaken and avoid the immune system at any cost in an event called immune evasion-which is regarded as a benchmark of cancer progression [219]. Immune evasion poses a solid interruption in finalizing the available anti-tumor therapies by resulting in an overall dysfunctional immune system with a subsequent loss of cell immunogenicity in tumor microenvironment (TME) [220]. Metastasis of the tumor is thus carried forward by such interactions between tumors and immune cells in the TME [221]. Blocking the immune checkpoints-a stretch of immune inhibitory pathways involved in the immune system may regulate the retention of the immune cells as a renewed principle for anti-tumor therapy [222].

miRNAs have been found to be deregulated in a number of anti-tumor therapies focused on challenging immune evasion by circular RNAs. For example, in a study by Wu et al. Hsa_circ_0020714 was promised as an immunotherapeutic agent as it regulates the miR-30a-5p/SOX4 axis in NSCLC [223]. Gao et al. suggested targeting circASCC3 as an immunotherapeutic agent as it sponges miR-432-5p to suppress anti-tumor activities in NSCLC [224]. TGFβ induced miR-183 repression of MICA/B expression also causes immune invasion and is thus posed as an immunotherapeutic target as well [225]. Although, none of the miRNAs mentioned above corresponds to the shortlisted core cell cycle regulating miRNAs, it is a promising field for further research.

## 7 Pre-clinical studies of miRNA-based therapy in lung cancer

As it has already been observed that miRNA plays an effective role in cell cycle regulation either as oncogene or tumor suppressor gene, several studies involving the manipulation of dysregulated miRNAs have been undertaken to determine the efficacy of miRNAs therapeutics in the treatment of lung cancer. However, the promising outcomes of those *in vitro* and *in vivo* experiments suggest that miRNA-targeted therapies could be a potential anti-cancer treatment strategy and thus provide the rationale for conducting this approach in human clinical trials. In this review article, we have focused on the therapeutic application of miRNAs which are directly involved in the manipulation of key regulators of lung cancer cell cycle (Table 5).

**Table 5:** miRNA based Therapeutic Strategies for Lung Cancer Treatment.

miRNA	Target	Model	Therapy types	Cancer	Delivery media	Function	References
miR-497	Cyclin E1	microfluidic 3D lung cancer model	Replacement therapy	Non-small cell lung cancer (NSCLC)	Exosome	Tumor suppressor	[234]
miR-29b	CDK6	A549 cell line and xenograft murine model	Replacement therapy	NSCLC	Cationic Lipoplexes	Tumor suppressor	[235]
miR-708-5p	p21 (cytoplasmic)	Xenograft mouse model	Replacement therapy	Lung cancer	polyethylenimine (PEI)	Tumor suppressor; Anti-metastatic	[124]
miR-34a and let-7	CDK6 and MYC	*Kras*-activated autochthonous mouse model	Replacement therapy	NSCLC	Neutral lipid emulsion	Tumor suppressor	[239]
miR-34a	CDK4	Subcutaneous H460 cell line and mouse model	Replacement therapy	NSCLC	MaxSuppressor in vivo RNALancerII (lipid based)	Tumor suppressor	[240]
let-7b and miR-34a	p53	Mouse model	Replacement therapy (Combinatorial therapy)	NSCLC	Lipid based delivery agent NOV340	Tumor suppressor	[247]
miR-208a	p21 (nuclear)	A549 cell line and H1299 cell line	miRNA inhibition	NSCLC	Exosome	Tumor suppressor	[250]
miR-143 and miR-506	CDK1 and CDK4/6	A59 cell line	Replacement therapy (Combinatorial therapy)	NSCLC	Lipofectamine 2000	Tumor suppressor	[249]
let-7a-5p	CDC25A, CDK6, and Cyclin D2	LSL-Kras-G12D mice	Replacement therapy	NSCLC	Lentiviral	Tumor suppressor	[251]
let-7b-5p	CDC25A, ​CDK6, and Cyclin D2	Subcutaneous H460 cell line	Replacement therapy	NSCLC	Local delivery	Tumor suppressor	[161]
miR-34a-5p and miR-124-3p	cMET and CDK6 for miR-34a-5p; STAT3 and ABCC4 for miR-124-3p	Intravenous A549 cell line	Replacement therapy (Combinatorial Therapy)	NSCLC	in vivo-jetPEI	Tumor suppressor	[252]
miR-154	Cyclin D2	A549 and Female BALB/c mice	Replacement therapy	NSCLC	Lipofectamine 2000 reagent	Tumor suppressor	[253]
miR-29b	Cyclin E1	A549 and H1299 cells	Replacement therapy	NSCLC	multifunctional micellarnanosystems (P103-PEI-RA/miR-29b)	Tumor suppressor	[254]
miR-613	CDK4	xenograft nude mouse mode	Replacement therapy	NSCLC	indicated plasmid DNAs	Tumor suppressor	[112]
miR-545	Cyclin D1 and CDK4	Nude mouse xenograft model.	Replacement therapy	Lung cancer	Lipofectamine 2000	Tumor suppressor	[226]
miR-365	CDC25A	mouse	Replacement therapy	NSCLC	Lipofectamine 2000 (Invitrogen)	Tumor suppressor	[237]
miR-3607-3p	Cyclin E2	Nude mice model	Replacement therapy	NSCLC	Lipofectamine 2000	Tumor suppressor	[255]
miR-449	CDK6, CDC25A	H1299 cell line	Replacement therapy	NSCLC	LipofectamineRNAimax	Tumor suppressor	[256]
miR-125b	p53	A549, H460, SW1573, LXF-289 cell lines	miRNA inhibition	NSCLC	Lipofectamine 2000	Oncogenic	[257]
miR-197	p53	A549, Calu-1	miRNA inhibition	NSCLC	LNAs (locked nucleic acids)	Oncogenic	[114]
let-7a	Cyclin D1	A549 and H1299	Replacement therapy	lung adenocarcinoma	lipofectamine 2000	Tumor suppressor	[229]
miR- 146a- 5p	Cyclin D1 and Cyclin D2	Xenograft mouse models	Replacement therapy	NSCLC	pRL vector	Tumor suppressor	[232]
miR-212/132 cluster	p21 and CyclinD1	A549 and H1299	Replacement therapy	Lung cancer	pLMP retroviral vector	Tumor suppressor	[246]
miR-503-3p	p21, CDK4	H292, H358 and H1975	Replacement therapy	NSCLC	pmirGLO Vector	Tumor suppressor	[245]
miR-137	CDK6	Xenograft mouse model	Replacement therapy	NSCLC	Recombinant plasmid eukaryotic expression vector (pcDNA3.1–CDK6)	Tumor suppressor	[236]
miR-224	p21 (nuclear)	Athymic BALB/c nude mice	miRNA inhibition	lung adenocarcinoma	pGCMV vector	Oncogenic	[117]
miR-25	cyclin E2,CDK2	H510A cells	miRNA inhibition	SCLC	Lipofactamine 2000	Oncogenic	[5]
miR-486-5p	CDK4	H1299 and SPCA-1	Replacement Therapy	NSCLC	Lipofectamine 2000	Tumor Suppressor	[227]
miR-330	E2F1	A549 cells	Replacement therapy	Lung cancer	GFP-pCMV-miRNA-330 vector	Tumor Suppressor	[118]
miR-433	E2F3	A549 and H460 cells	Replacement therapy	NSCLC	Lipofectamine 2000	Tumor suppressor	[120]
miR-29a	Cyclin D1	A549 cells	Replacement therapy	Lung cancer	PEN/miR-29a nanoparticles	Tumor Suppressor	[230]
miR-193a-3p	Cyclin D1	A549, H1299, H1975, H460 cells	Replacement therapy	NSCLC	Lipofectamine RNAiMax (Thermofisher)	Tumor Suppressor	[7]
miR-193a-3p	Cyclin D1, CDK2 and CK6	A549 cells; mice	Replacement therapy	Lung cancer	Lipofectamine RNAiMAX; C12-200 lipid nanoparticles	Tumor Suppressor	[231]
miR-122-5p	p53	A549 cells	miRNA inhibition	NSCLC	Plasmid	Oncogenic	[122]
miR-660	p53	SCID mice	Replacement therapy	NSCLC	Coated Cationic Lipid-nanoparticles (CCL)	Tumor Suppressor	[244]
miR-150	p53	A549 cells and nude mice	miRNA inhibition	Lung cancer	PR-ASO-150 vector	Oncogenic	[241]
miR-34	p53	A549 cell line	Replacement therapy	NSCLC	PAM-Ap/pMiR-34a	Tumor suppressor	[243]
miR-155	p53	Orthotopic lung cancer model	miRNA inhibition	Lung cancer	Lentiviral Vector	Oncogenic	[54]

### 7.1 Preclinical studies related to miRNA replacement therapy

Numerous studies have been done to investigate the role of miRNA replacement therapy. Early work had shown that, miR-545 levels in lung cancer tissues are downregulated and it may act as tumor suppressor sincemiR-545 mimic inhibits the cell cycle in G0/G1 phase by repressing the expression of Cyclin D1 and CDK4 genes [226]. MiR-486-5p and miR-613, both target CDK4, have been shown to inhibit NSCLC cell progression and might be used as a tool for miRNA replacement treatment [227, 228]. In addition, Zhao et al. found that the expression of let-7a is downregulated in lung adenocarcinoma and transfection of let-7a mimic into A549 and H1299 cells inhibits cell proliferation by decreasing the expression of Cyclin D1 but increased the expression of Rb. Furthermore, they also conducted transwell experiments and the result indicated that let-7a inhibits migration and invasion of lung cancer cells by influencing cyclin D1 [229]. Another experiment was conducted in lung adenocarcinoma cell line to evaluate the role of miR-29a in cell progression, migration and invasion. In this analysis, a polyethylenimine (PEI) derivative, Nisopropylacrylamide-modified PEI (namely PEN) was constructed as a stable carrier of miR-29a mimic to protect against the nuclease degradation. The PEN-mediated miR-29a transfection showed effective inhibition of cell migration and invasion as well as its anti-proliferative effect by decreasing CyclinD1, a key regulator of the G1/S transition [230]. The anti-tumor function of miR-193a-3p has been established through multiple *in vitro* and *in vivo* experiments. In a recent study, miR-193a-3p mimic has been formulated in a novel lipid-based nanoparticle, named INT-1B3 which consistently suppresses some pro-tumorigenic phenotypes in lung cancer cell lines by downregulating the expression of oncogenic Cyclin D1 [7]. In another study, the tumor suppressor activity of miR193a-3p mimic has been evaluated using systemic administration of C12-200 lipid nanoparticles containing miRNA mimic and the experiment concludes with the anti-proliferative and apoptotic properties of this mimicmiRNA by targeting cell cycle proteins. The. long-term effect of this replacement therapy has also been tested on normal cells but no detectable changes on proliferation as well as apoptosis has been observed [231]. The overexpressing miR-146a-5p (pLenti-miR-146a-5p) has shown antitumor effects by substantially downregulating two central cell cycle regulators, namely CyclinD1 and CyclinD2 [232]. Trang et al. conducted an experiment in murine models to investigate the impact of let-7 miRNA, as miRNA replacement therapy against lung cancer formation. In their study, lentivirus expressing let-7a was administered intranasally into six-week-old LSL-Kras-G12D mice whereas H460 cell lines were transfected with local delivery of synthetic let-7b by intratumoral injections [161]. Though both delivery routes of action have shown the effective remission of lung tumors in NSCLC mouse model, a great attention should be given in delivery technology for efficacious clinical application. Furthermore, transfection of miR-154 into NSCLC A549 cells is able to decrease cell proliferation, invasion, and migration and increase cell apoptosis and halt cell cycle at G0/G1 stage, as well as inhibit tumor growth *in vivo* mice model [149]. In two different studies, miR-29b and miR-497 mimic have showed anti-tumor activity by significantly reducing the expression of cyclin E1, however the delivery strategies differ. Codelivery of miR-29b mimic and Retinoic Acid (RA) into NSCLC cells using multifunctional polyethylenemine based micellarnano-systems, named P103-PEI-RA/miR-29b (10/1) showed more significant therapeutic effect than individual miR-29b mimic delivery. In addition, the developed version of micelleplexes showed the ability to maintain their structure during the delivery of encapsulated RA and miR-29b by avoiding unspecific interactions with serum proteins. Although this innovative delivery process of miRNA showed a successful result for NSCLC cell lines, *in vivo* analysis is required to validate this proceeding into clinical trial [233]. In contrary, Jeong et al. developed miR-497-loaded exosomes as a potential therapy for the treatment of NSCLC, based on the fact that miR-497 has anti-angiogenic and anti-tumor effects by suppressing the expression of Cyclin E1 (CCNE1) along with HDGF (Hepatoma-derived growth factor), YAP1 (Yes-associated protein 1), and VEGF-A (Vascular endothelial growth factor-A). Inthe study, exosomes were considered as a delivery vehicle since they provide stable protection and effective delivery of miRNA. Thus, this complex exosome-mediated miRNA therapeutics exhibited decreased level of tumor growth, migration and angiogenesis both in A549 and a microfluidic 3D NSCLC model [234]. Transfection of miR-3607-3p antagomiR into nude mice against CyclinE2 has resulted in low level of tumor development and metastasis through G0/G1 arrest and S phase reduction. This miRNA has also been identified to play a role in the pathogenesis of NSCLC through signaling pathways [123]. A cationic lipoplex containing miR-29b (LP-miR-29b) has been successfully delivered to A549 cells and xenograft murine model and in both cases, it caused reduced amount of tumor growth as well as clonogenicity by inhibiting the expression of CDK6. In the study, they more over show that LP-miR29b is more efficacious in delivery process, tumor growth suppression and also sensitivity to the chemotherapeutic agent cisplatin than NeoFX-miR29b. Remarkably, this lipid based nanoparticle has not shown any notable cytotoxic effects on the major organs of mice, suggesting that miR-29b has minimal off-target effects [235]. Zhu et al. found that the restoration of miRNA-137 in lung cancer cells causes lower expression of CDK6 at both the mRNA and protein level, resulting in G1 cell cycle arrest as well as cell death *in vitro* and *in vivo*. They also explored the application of 5-aza-2′-deoxycytidine, a DNA methylation inhibitor, and Trichostatin A, a histone deacetylase inhibitor, in NSCLC cell lines considering the fact that miR-137 has been found to be epigenetically suppressed in other tumors [236]. The significant function of those DNA-hypomethylating agents in the increased expression of miR-137, suggesting combination of miRNA and epigenetic treatment may act as an effective therapeutic approach for lung cancer. Restoration of miR-365 has shown its inhibitory function on tumor growth as well as high sensitivity effect to radiotherapy through down-regulation of CDC25A. The combination of miR-365 along with local IR has shown substantially greater effect on the reducing xenograft tumor size than treatment with local IR individually, suggesting a promising therapeutic strategy for NSCLC [237]. In lung cancer H1299 cells, transfection of miR-449a/b has resulted in oncogenic CDK6 and CDC25A to be suppressed, leading to the dephosphorylation of pRB and decreased amount of E2F1 expression [238].

To date, most of the studies have been done investigating the effects of miR-34 in NSCLC treatment due to its activity as a master tumor suppressor by silencing its multiple targets (e.g. CDK4, CDK6) involved in cell cycle regulation. Wiggins et al. and Trang et al. have investigated the delivery of the miR-34a mimic using a neutral lipid emulsion and both approaches resulted in effective inhibition of lung tumor growth [239, 240]. In addition, Trang et al. have also explored the efficacy of let-7 mimic along with miR-34a as both of them share a few similar targets. In the work, miR-34 mimics led to reduced proliferation and higher apoptosis, whereas let-7 mimics merely resulted in lower proliferation. It suggests that a combination of let7/miR-34 may provide better therapeutic benefits than any of the mimic used alone [239]. Furthermore, Wiggins et al. has also observed that this delivery strategy does not cause an elevation of liver and kidney enzymes in serum, nor does it induce a non-specific immune response in mice, indicating that it may be less cytotoxic than cationic lipid emulsion [240]. Restoration of reduced miR-330 and miR-433 suppresses the proliferation and invasion of lung cancer cells by targeting the two members of transcription factor family, E2F1 and E2F3, respectively [118, 120]. However, increased expressions of apoptosis markers, such as caspase-3 and caspase-9 protein, and decreased expressions of cell migration markers, such as C-X-C chemokine receptor type 4, vimentin, and matrix metalloproteinase 9, have been observed after stable transfection of PCMV vector containing miR-330 into cancerous cell lines, making this miRNA a great candidate for miRNA replacement therapy in lung cancer.

### 7.2 Preclinical studies related to miRNA inhibition therapy

Antisense oligonucleotides, also known as antimicroRNAoligonucleotides, have been found to be exceptionally effective at inhibiting the action of oncogenic miRNAs that are upregulated in lung cancer. Intratumoral delivery of an anti-miR-150 expression vector (PR-ASO-150) in nude mice has shown suppressive action on overexpressed miR-150, causing reduced amount of tumor volume and weight by contributing to upregulation of apoptotic gene p53. In that study, they have also demonstrated that the percentage of apoptosis is higher (28.99%) in PR-ASO-150 treated group than the control group (3.38%) [241]. In a different study, Almost a two-fold increased level of p53 protein has been reported in NSCLC cell lines resulting in decreased cell survival after treatment with miR-125b antagomiR [242]. However, LNA-197 (locked nucleic acids) has proven its neutralization activity against oncomiR miR-197and thus induced higher levels of p53 [166]. By knocking down microRNA-122-5p, researchers have been able to diminish p53 mRNA and protein levels, as well as arrest the cell cycle in the G0/G1 phase and reduce cell migration. Additionally, this reduction is related to the decreased level of AKT and PI3K phosphorylation [122]. Transfection of antisense oligonucleotide of miR-25 has downregulated the overexpressed miR-25 in SCLC cell line, resulting in decreased amount of cancer cell proliferation, invasion, and resistance to cisplatin by suppressing the expression of CyclinE2 and CDK2.

Two different but effective delivery strategies, the PAM-Ap/pMiR-34a and the Coated Cationic Lipid-nanoparticles entrapping miR-660 (CCL660) have been found to contribute in inhibition of lung cancer cell proliferation, migration, invasion and promoting apoptosis by restoring the activity of p53 [243,244]. Aptamer conjugated nano-complex containing miR-34a has shown protective effects from the degradation in serum for 48 h and exhibited significantly higher (7.93 fold) amount of p53 mRNA than that of PAM/pMiR-34a (2.1 fold) [243]. However, neither the acute inflammatory cytokines nor the pro-inflammatory cytokines have been found to be modulated after single or long-term administration of Lipid-nanoparticles containing synthetic miR-660, demonstrating its non-toxic effects, whereas it only has reached into 30% of lung cancer cells in a mouse model, showing its low delivery efficiency [244]. This limitation may be solved by adding a tumor cell-specific ligand on the surface of lipid nanoparticles to strengthen the therapeutic efficacy of miR-660.

p21, a p53-inducible protein has been directly targeted by miR-708-5p, miR-208a, miR-224 and miR-503-3p. Interestingly, p21 shows divergent function either as an oncogene or a tumor suppressor gene depending on its cellular localizations and the conditions. Based on this dual role of p21, miRNA replacement or inhibition therapy has been considered for lung cancer therapy. The systematic delivery of linear PEI/miR-708-5p mimic complexes has demonstrated anti-metastatic and tumor suppressor roles in lung cancer mouse model, with lower levels of cytoplasmic p21 (oncogene) and inactivated PI3k/AKT pathway [124]. By suppressing the expression of p21 and CDK4 mRNA at the same time, miRNA-503-3p mimic has been shown to function as a tumor growth inhibitor and apoptosis mediator [245]. On the other hand, transfection of exosome mediated miR-208 inhibitor into NSCLC cell lines and pGCMV vector containing anti-miR-224 into nude mice have both shown cell cycle arrest through the upregulation of p21 mRNA and protein [81, 117]. As cytoplasmic p21 promotes antiapoptotic actions and nuclear p21 is associated to cell cycle arrest, localization of p21 should be taken into consideration while miRNA based therapeutic approach shouldbe contemplated to target p21. Furthermore, transfection of antisense oligonucleotide of miR-25 has downregulated the overexpressed miR-25 in SCLC cell line, resulting in decreased amount of cancer cell proliferation, invasion and resistance to cisplatin by suppressing the expression of cyclinE2 and CDK2 [5]. Mature miR-132 and miR-212 exhibits similar sequences and share the same seed regions for target mRNA and based on this ‘dual targeting’ capability, pLMP-miR-212/132 has been constructed to investigate its effectiveness in lung cancer therapy. However, overexpression of this cluster miRNA has been found to exhibit radio-sensitivity and induce cell cycle arrest at the G1/S phase by increasing the expression of p21 while decreasing the expression of Cyclin D1 [246]. Tumors exposed to the combinatorial therapy of let-7 and mir-34 have shown greater reduction in tumor volume and cell invasion with no accumulation of serum cytokines such as tumor necrosis factor, interleukin-6 and interleukin-1b in NSCLC mouse model than the tumors treated with each miRNA alone [247]. The introduction of nCAR assembled bioengineered miR-34a-5p and miR-124-3p into human lung carcinoma cell *in vitro* and in xenograft mouse models *in vivo* has demonstrated the selective release of target miRNAs into lung carcinoma cells, inhibiting the proliferation and migration through specific regulation of target gene expression [248]. In NSCLC cell lines, a substantial apoptotic response and potential antiangiogenic efficacy of miR-143 and miR-506 combinatorial therapy has been found, which also induces cell cycle arrest at the G1/S and G2/M phase transition by suppressing Cyclin Dependent Kinases (i.e., CDK1, CDK4 and CDK6) [249].

## 8 Clinical studies

Although the emergence of miRNA therapeutics in clinical development of lung cancer is still in its budding stage, the success rates in pre-clinical studies are showing its brightening site. Some miRNA-based drugs have proceeded into the phase I clinical trial, however several challenges do not allow them for FDA approval. Putative miRNA drugs have exhibited to be more effective in other clinical studies rather than trials for lung cancer. From that context, we have attempted to emphasize the studies related to research and advancements of miRNA-based treatment in lung cancer in order to promote the field a bit deeper.

MRX34, the most advanced compound entered into first-in-human multicenter phase I trial in patients with small cell lung cancer along with melanoma, multiple melanoma, lymphoma, liver cancer or renal cell carcinoma in 2013. In 2016, the trial included a total of 99 patients with advanced solid tumors, including NSCLC patients. This trial involved a dose-escalation study with intravenous infusions two times per week for a portion of the population and five times per day for the rest. At the end of the trial, some patients had shown positive effects of MRX34 whereas 96% of all patients had experienced toxic effects of immune response. The trial, however, was terminated due to the death of four patients [258]. Final study of this trial included the intravenous administration of MRX34 5 days in 3-week cycles, with oral dexamethasone premedication as the recommended Phase 2 dose (RP2D) which demonstrated a tolerable pattern of toxicity in some individuals. Reduced amount of platelets, complement activation, and higher organ weights due to macrophage hypertrophy were the major dose-dependent effects identified in these studies [259]. Despite the small number of lung cancer patients included in this study, MRX34 has the potential to be a therapeutic compound for lung cancer once the drawbacks are addressed. For this purpose, pre-clinical trials will need to be re-designed, with a particular emphasis on immune-related toxicities and specific tissue-based delivery of these constructs. In addition, different dosing schedules and pre-medication regimens must be investigated. There is currently no more MRX34 research being undertaken or planned; nevertheless, there are still many possibilities for re-investigation. In a phase I clinical study, TargomiRs has exhibited promising outcomes in Advanced Non-Small Cell Lung Cancer patients with no adverse immunological effects. This drug consists of a miR-16-based microRNA mimic, bacterial minicells based EnGeneIC Delivery Vehicle (EDV) and targeting moiety (i.e., bispecific antibody) that recognizes a specific protein on target cells [260]. The antimiR against oncomiR miR-122 has been studied in a preclinical phase and has shown to be effective in lung cancer cell lines [230]. Though this study has not proceeded into clinical trial, RG-101 (N-acetyl-D-galactosamine)-conjugated antimiR against this miRNA, has undergone phase I trials in HCV infected individuals [261]. A phase II study is currently being conducted where RG-101 is combined with direct-acting antivirals such as Harvoni, a combination of ledipasvir and sofosbuvir drugs. This study result has shown a 100% response rate with no relapse at 24 weeks [262]. As a result, this antimiR could be investigated in lung cancer clinical trial, either alone or in conjunction with other drugs, to up-regulate the expression of the p53 regulator of cell cycle utilizing a targeting moiety, as it has previously been demonstrated to be effective in other cancer treatments. Currently, a medicinal product INT-1B3 has entered into its Phase I clinical trial (NCT04675996) in the United States and Europe expecting up to 80 patients to be enrolled. INT-1B3 is a lipid nanoparticle containing miR-193a-3p mimic designed to target multiple solid tumors including lung cancer. The Phase I study is a dose-escalation phase which is mainly destined to determine the safety profile of INT-1B3 as well as its maximum tolerated dose for recommending the dose for phase 2. This Phase Ia trial is expected to be completed in the first half of 2022. Subsequently, the pharmacokinetics, pharmacodynamic response and antitumor activity of this miRNA mimic-based INT-1B3 will be evaluated in Phase Ib study [56].

Although miRNA has not been yet confirmed or authorized for lung cancer treatment, its clinical applicability can be explored in a sequential way from target identification to therapeutic approach (Figure 3a).

## 9 Limitations & future prospects

Given the stability and steadiness in serum and plasma rather than those of mRNAs, it can be decided that miRNAs are rightfully justified to be elected as non-invasive biomarkers for tracing disease progression and eventually cancer classification. In fact, as we know, many miRNAs are deregulated in multiple cancers and they correspond to a wide array of target genes involved in the oncogenic pathway. So, for diagnostic and monitoring purposes, utilizing miRNAs may seem to be a formidable choice. Even for a treatment option, miRNA (or anti-miRNA) therapeutics have been much popularized and emphasized over the last decade or so [263]. However, the complexity through which the miRNAs operate, non-specific side-effects are bound to follow [264].

In case of lung cancer therapy using miRNAs, certain limitations have been accompanying the process inevitably as well. For one, miRNA and anti-miRNA therapeutics applied in cases of lung cancer are too limited. Only a handful of initiatives such as- Targo-miRs (MesomiR 1) and MRX34 could enter the clinical trials in patients with NSCLC so far [265]. Even so, MRX34 had to be terminated at phase-I due to multiple adverse immune responses in the NSCLC patients whereas Targo-miRs made it past phase-I clinical trial [266]. No trial of miRNAs regulating the tumor microenvironment (TME) of lung cancer has been developed yet. Also, a lack of reproducibility can be observed among the studies dedicated to diagnosis of lung cancer through miRNAs either due to the insufficient case numbers in large scale projects, variation of techniques followed within, and inconsistencies of serum miRNA level across the individuals tested [267]. miRNA delivery systems have just as much importance as the miRNAs themselves and thus can be an issue of rising obstacles as well. For example, clinical use of viral vectors for miRNA delivery has been discouraged [268]. Because, the most familiar viruses used in this sense such as Retro and Adeno viruses, despite having reduced replication ability and higher transfer rates of miRNAs, are suspected of toxin production, eliciting immune response leading to inflammatory reactions, and causing mutation within the host. The non-toxic vectors such as polymer-based and liposome mediated ones show an opposite effect in this regard as in exerting lower toxicity but working with a much lower delivery rate. However, LNA is the safest and most successful method so far which is still being researched [269, 270]. In addition, some studies showed promising result by manipulating the expression of specific miRNAs that can alter the drug sensitivity or radio sensitivity.

## 10 Conclusion

From our review, we have highlighted several miRNAs having the potential to exhibit sensitivity to current lung cancer therapy. These miRNAs may be further investigated along with the combination of radio or chemotherapy for establishing a more effective therapeutic approach against lung cancer. Lung cancer is one of the most concerning malignances in the world leading to a bulky chunk of cancer-associated deaths all over the world. Prime setbacks in achieving success in lung cancer treatment include deficiency in early detection methods and a widespread acquisition of drug resistance [89]. In search of a novel solution to such a problematic development, many approaches have been proposed overtime including consideration of miRNA-based therapeutics. Hence, we have accumulated varying information on miRNA engagement in lung cancers from different sources and attempted to take this trend into a newer direction. By listing out only the miRNAs dysregulating the core cell cycle regulators, we have reduced our large pipeline for miRNA research into a selective but effective set of candidates and zoomed in on each of the prospects for figuring their respective biological roles in lung cancer, their eligibility as biomarkers in prognosis or diagnosis of lung cancer, usability as therapeutic plans and their response mechanisms against radio or chemotherapy for a detailed but precise understanding that might help materialize the ideas into a medical revolution. With the emergence of Systems biology a deeper understanding of the miRNA-mediated gene regulation network would assist in this regard in pushing through safer miRNA therapeutics to the clinics.

## Declarations

### Author contribution statement

All authors listed have significantly contributed to the development and the writing of this article.

### Funding statement

This research did not receive any specific grant from funding agencies in the public, commercial, or not-for-profit sectors.

### Data availability statement

Data included in article/supp. material/referenced in article.

### Declaration of interests statement

The authors declare no conflict of interest.

### Additional information

No additional information is available for this paper.
